# Health-Related Quality of Life in Children with Celiac Disease: A Study Based on the Critical Incident Technique

**DOI:** 10.3390/nu5114476

**Published:** 2013-11-12

**Authors:** Chiara Biagetti, Giulia Naspi, Carlo Catassi

**Affiliations:** Department of Pediatrics, Università Politecnica delle Marche, Via Corridoni 11, Ancona 60123, Italy; E-Mails: giulia.naspi@live.it (G.N.); catassi@tin.it (C.C.)

**Keywords:** quality of life, celiac disease, gluten free diet, children, lived experiences, psycho-social aspects

## Abstract

Celiac Disease (CD) is a chronic autoimmune disease triggered by dietary gluten. Gluten avoidance, which is the only available treatment for CD, could impact on quality of life of children with CD. We present the results of a qualitative study on the emotional impact of gluten free diet (GFD) on the everyday life of children affected with CD. We investigated 76 celiac patients aged 2–18 years (average age: 9.5 years). By using the Critical Incident Technique (CIT), we defined emotions related to difficulties and awkward situations experienced by the patients. Written answers to open-ended questions from either children (older than 8 years) and parents (children younger than 8 years) were analyzed qualitatively. We found 80 dilemmas experienced in three different arenas (food situations at school, meals at home, meals outside) and characterized lived experiences of children with CD in everyday life (specific emotions, difficulties in relationships and in management of daily life). Children with CD experience strong emotions related to the GFD, permeating several aspects of everyday life. These dilemmas may be missed by a conventional, questionnaire-based approach to the psycho-social consequences of CD treatment.

## 1. Introduction

Celiac Disease (CD) is a permanent autoimmune disorder triggered by dietary gluten in genetically predisposed individuals. It is a frequent condition affecting about 1% of the general population. Gluten is a protein complex found in cereals that are diffusely consumed in most countries, *i.e*., wheat, rye and barley. The only treatment that is currently available for CD is the lifelong avoidance of gluten from the diet (so called gluten-free diet = GFD). This treatment is effective but limits the patients’ food choice and influences the patient’s lifestyle and quality of life (QOL) [1].

During the last 10 years the interest on how patient perceive the impact of chronic diseases and how the health state is modified by the therapeutic intervention is getting increasing attention in medical and health care settings. Health-Related QOL (HRQOL) is a multidimensional concept including physical, emotional, social and cognitive domains. What matters in HRQOL is the way patients feel about their functioning, not their functioning itself [2].

Few data are available about QOL of children on GFD. Dietary restrictions can be difficult to accept and follow, especially for screening-detected patients and during adolescence, a life period characterized by lower adherence to the dietary treatment [3]. Nevertheless recent studies suggest that adolescents with screening-detected CD have similar HRQOL as their peers without CD, both at diagnosis and after one year of treatment [4]. A study on the long-term health and QOL after mass-screening for childhood disease reported a similar QOL after 10 years follow-up between children on GFD and controls [5]. Early CD diagnosis seems to be associated with better physical health, lower CD-associated burden and fewer social problems [6]. Other studies used disease-specific questionnaire in order to elicit specific problems related to the GFD. In 2001 *Kolsteren et al*. reported that the QOL of children with CD was similar to that of an healthy age-matched population using the TACQOL-COE-DIET (Technisch Natuurkundig Onderzoek-Academisch Ziekenhuis Leiden Children’s Quality Of Life Celiac Disease Gluten Free Diet) questionnaire [7]. Using the CDDUX (Celiac Disease Dutch Children) quality of life questionnaire, CD children had a lower QOL than the healthy reference group. The CDDUX tool is a “bottom-up” questionnaire developed using a focus group-based approach that allow to perceive the situation from the child point of view; this kind of questionnaire could have elicited more specific information about those aspects of life that are typically influenced by the disease [8,9].

The variability of results yielded by different questionnaires and the insufficient data about QOL in children with CD need further investigations.

An alternative method of investigating the QOL is based on the Critical Incident Technique (CIT), that is a qualitative research tool (open-ended questions). Studies in adults with CD showed that negative lived experiences and feelings of adults with celiac disease occur frequently and have an impact on daily life. Patients’ “dilemmas” are related to awkward situations or the need to cope difficulties in social relationships and management of daily life in order to adhere to GFD, as reported by Sverker and colleagues [10]. Given the interesting results reported in adults with CD by using the CIT approach, in the present study we aimed to investigate the impact of CD and the GFD on the HRQOL and the social and emotional world of children with CD, using this qualitative method of research.

## 2. Experimental Section

### 2.1. Patients

Eighty children (aged 2–18) with biopsy-proven CD on the GFD by at least one year were invited to participate in the study when seen at the Gastroenterology Outpatient Clinic (Department of Pediatrics, Università Politecnica delle Marche, Ancona, Italy) for follow up, between 2006 and 2009. To assess the compliance to the GFD, determination of CD serological markers was performed in the three months preceding the enrolment in this study. Children under 2 years and/or affected by type 1 diabetes (T1D) were excluded. Children with CD showing elevated levels of serological markers were included in the study. Parents were informed about the study’s risks and benefits and agreed to participate through a written informed consent. The study protocol was approved by the Ethical Committee of the Università Politecnica delle Marche, Ancona, Italy.

### 2.2. Instrument

We used the Critical Incident Technique (CIT), a qualitative research instrument representing a practical and structured way of collecting and analyzing information about human activities and their significance to the involved individuals. This tool is useful to yield rich, contextualized data that reflect real-life experiences in a flexible way, to meet the specific situation at hand [11]. It requires collection of brief, written, factual reports of “critical” actions or experiences in response to explicit situations or problems in defined fields. In this study we defined a “critical incident” as a “perplexing or awkward situation perceived by a patient to cause disturbances in his/her everyday life” [10]. With this phenomenological approach, the goal of the study is to define the individuals’ own world. The method is sensitive to minor problems that are however important for the individual (“dilemmas”). We collected dilemmas until redundancy appeared, in order to carry out a meaningful qualitative analysis as suggested by Sverker and co-workers [10].

We chose to collect data through written answer to the following open-ended question:

▪ Could you describe the last occasion in which you thought: “If I were not affected by CD” (children older than 8 years)
▪ In your opinion what are the major difficulties your child had to cope while following the GFD? (parents of children under 8 years)

A sheet reporting the questions was delivered to patients (children older than 8 years) or parents (children younger than 8 years). They were invited to write their answers. The interviews were administered at the end of the follow-up visit, in a quiet and empathetic environment. Participants were leaved alone during the test and the interviewer came back at the end of the test; at this time answers were read aloud and sometimes the interviewer asked follow-up questions, to help the youngest patients if their answers were not precise, not tying themselves to a specific incident, or to better understand the single situation or experience, or to know other experiences or dilemmas perceived by the patients, all related to CD and the GFD. All the answers to the follow-up questions were then written by the participants.

### 2.3. CIT Data Analysis

To analyze the data obtained by the CIT we used the method previously described by Sverker *et al*. [10]. First the examiner read several times each written answer, in order to become familiar with the data. From each interview the examiner abstracted one or more dilemmas and listed them in different categories. Some informants described more than one critical incident and identified dilemmas were listed in three main categories. The next step was to find an appropriate label for each dilemma and category; then the examiner identified three arenas in which dilemmas were experienced. An initial classification was presented by the first author (C.B.) and then discussed and revised within the research group.

## 3. Results

### 3.1. CIT Interviews

Eighty children were initially screened. Three patients were excluded because of lack of parental consent and one patient was excluded because of co-morbidity (T1D), so seventy-six children agreed to participate and were included in the study. Only two patients had abnormal values of the serological CD markers. The demographic characteristics of the study group are described in Table 1. Twenty-one patients did not report difficulties related to the GFD, 11 out of 33 symptomatic patients (33%), and 10 out of 43 asymptomatic patients (23%) (*p* = 0.3, chi square test).

**Table 1:** Main characteristics of study group.

	Patients with CD (*n* = 76)
**Age (years)**	
median (95% CI)	8.7 (7.4–9.9)
25th–75th percentile	6.1–12.8
**Age below 8 years**	
[*n* (%)]	33 (44%)
**Gender **	
Male [*n* (%)]	18 (23.7%)
**Associated disorders ^a^**	
[*n* (%)]	16 (21.1%)
**Age at CD diagnosis (years)**	
median (95% CI)	3.5 (2.6–4.4)
25th–75th percentile	1.9–7.0
**Duration of GFD (years)**	
median (95% CI)	3.9 (3.2–4.5)
25th–75th percentile	1.7–5.4
**Typical CD at diagnosis ^b^ [*n* (%)]**	33 (43.4%)
**No reported dilemmas related to GFD [*n* (%)]**	21 (27.6%)

^a^ Associated disorders were: mild allergic disorders (*n* = 10), asthma (*n* = 5), autoimmune thyroiditis (*n* = 2); ^b^ Typical CD: patients presenting with classical symptoms, *i.e*., abdominal distension, vomiting, diarrhea, weight loss.

**Figure 1:**
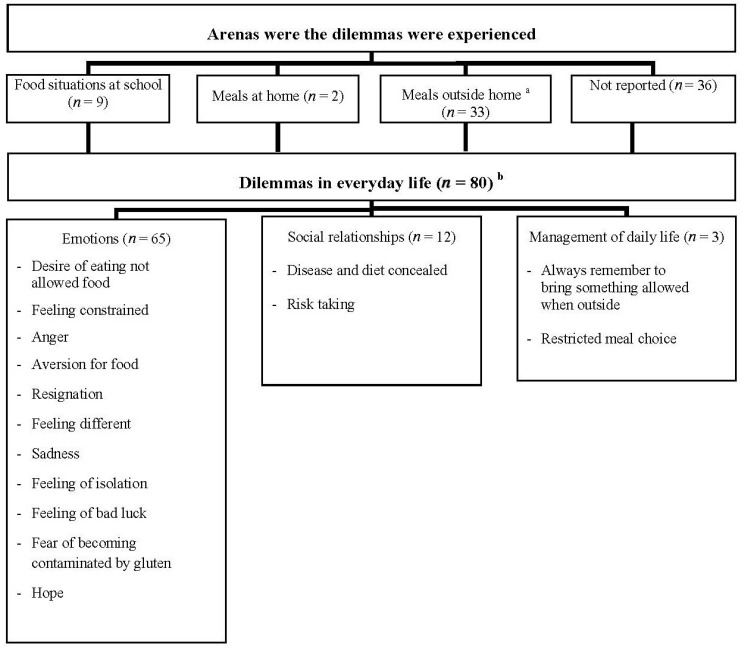
Lived experiences of dilemmas (*n*) in everyday life among children with celiac disease.

^a^ Meals outside home: supermarket, parties, restaurant, at relatives’ or friends’ home; ^b^ The number of dilemmas is higher than the number of children with celiac disease reporting critical incidents related to GFD (*n* = 55), since some patients reported more than one dilemma.

We collected a total of 80 dilemmas experienced by 55 patients, some patients reporting more than one dilemma. We identified three main categories of dilemmas (emotions, relationships and management of daily life) that patients experienced in three different arenas: (1) meals at school; (2) meals at home; (3) food situations and meals outside home (Figure 1).

We didn’t find any significant difference on dilemmas distribution between symptomatic and asymptomatic patients. There were no noticeable differences in reported dilemmas between parents and children.

### 3.2. Emotions

The lived experiences of children with CD were characterized by specific emotions, as summarized in Figure 1. The most frequently reported emotion was the desire of eating gluten containing foods that were not allowed; sometimes it was just to try, but often the desire raised just because it was forbidden to share or taste the same food that the parents or friends were eating. Sometimes this emotion was caused by avoidance of food that children liked very much before starting the GFD.

*“The most important difficulty my son meets is being not allowed to share the same “gluttonies” as their cousins do when we are outside home all together.” (5 years)*

What is interesting in children’s reports is the kind of adjectives used to describe food: the gluten containing items are “great”, “good”, “tasty”, while GFD is reported as “different”, “often crumbled”, “smelly”, “cold”.

Gluten avoidance was associated with specific moods, e.g., sense of restriction and constraint, sadness, anger, sacrifice and resignation:

*“My son doesn’t accept the imposition of “his food.” (7 years)*

*“When I can’t eat what my brother or my friends are eating at school parties, I get angry because what I can do is just see them eating their snacks or meals, but I can’t share. I don’t show my anger to my friends, I don’t want others know what I feel.” (8 years)*

*“Every time I see other children eating I get angry and I eat the same foods they are eating, or I go to the supermarket and I buy all the things I’d like to eat, without thinking about gluten content.” (13 years)*

*“Last Monday I went to the supermarket with my father and I had to give up a candy I liked very much.” (8 years)*

This diet restriction could lead to food refusal in youngers or conscious transgressions in adolescents. In particular adolescents sometimes chose to eat gluten containing food just to conceal their intolerance to their mates (see social relationship).

Children and adolescents reported to feel different when they could not do the same things in a peer group, since they had to eat something they brought or they had to “say no”. Sometimes isolation was a disappointing adults’ fault, because someone didn’t know appropriate strategies to cope with a child on the GFD.

*“I wished I wasn’t celiac when I went for a walk with my friends last Saturday. They bought hot pizza, while I had to eat my cold and* *‘different’ pizza, that I brought with me from home. If I were not a celiac I could eat the same pizza that my friends eat.” (10 years)*

*“When we eat at school, I have to sit alone in a different table; sometimes I think I have a bad luck because I can’t eat what my friends eat, while they can eat their food and also my food.” (9 years)*

Older children and adolescents were more frequently conscious of their intolerance and of risks related to gluten intake. They could experience fear of becoming contaminated by gluten:

*“It happened twice that my friends at school brought a slice of pizza near to my mouth for joke, and I had a great fear of getting ill for this.” (13 years)*

An interesting aspect of children’s world of lived experiences is a great hope for the future:

*“My daughter is hopeful and she’s waiting for new therapies.” (7 years)*

*“My son doesn’t accept his new diet and he hopes that when he will be older he will eat all kind of food.” (7 years)*

### 3.3. Relationships

GFD strongly influences social relationships. Children, particularly adolescents, did not want to reveal their food intolerance to their mates. They did not want their diet be the subject of conversations, because they did not want to feel different when they were in a peer group. For this reason they sometimes decided to eat gluten containing food consciously taking risks, while others chose not to go out with friends for lunch or dinner, to avoid the problem:

*“At school I don’t eat ‘my snack’ that my mother puts in my backpack because I don’t want my mates know about my gluten intolerance.” (10 years)*

*“Two weeks ago on Saturday I was at dinner with my mates: everyone had a pizza and I also had a pizza, not to be forced to answer questions about my diet and not to feel different.” (16 years)*

### 3.4. Management of Daily Life

The GFD and the related need to avoid some food from the diet necessarily impact on the management of daily life. In particular adolescents, more than children, reported practical difficulties related to the adherence to the GFD. Some adolescent described restricted meal choice when dining at restaurant or at the supermarket. Schools were often organized to offer something to eat, much less frequently offered gluten-free snacks. This situation was awkward for some and sometimes led to giving up going out for dinner or find alternative strategies, like always remembering to bring some gluten free food with them.

*“Last summer I was in a great bakery in Sicily; there were a lot of cakes, but I could only eat a little chocolate.” (12 years)*

*“Last week at school I forgot my snack; all my mates bought snack at automatic machines, but I couldn’t. I was really hungry!” (12 years)*

*“It was difficult to learn what kind of food I can buy at the supermarket and it is also really difficult to find new food at the supermarket, just to have different choices.” (12 years)*

## 4. Discussion

Most studies focusing on the QOL of children with CD or adolescents on treatment with the GFD reported no differences between patients and healthy reference groups. This “optimistic” view is in contrast with follow-up studies suggesting that the adherence to the dietary treatment is often poor, particularly in adolescents, as a consequence of the heavy psycho-social burden imposed by the GFD on daily life. Poor adherence to the treatment is likely to be the cause of the incomplete recovery of the small intestinal mucosa that is frequently found at follow-on intestinal biopsies [12]. In turn, persistent intestinal damage may cause long-term CD complications, such as osteoporosis and intestinal lymphoma [1]. In this study only two patients had abnormal levels of serological CD markers. We think that our sample of children with CD was somewhat selected because these patients regularly attended our Gastroenterology Outpatient Clinic. Furthermore it should be highlighted that minimal transgression to the gluten-free diet do not necessarily lead to abnormal antibody levels.

In our opinion questionnaires used to measure the QOL are not always the appropriate instrument to catch and characterize the psycho-social discomfort associated with the long-term dietary treatment of CD. By using a qualitative method of investigation (the CIT) we found that most children and adolescents (72%) reported and clearly described their difficulties in following the GFD. This method highlighted the specific problems related to the GFD and their emotional and social impact on everyday life of children and adolescents with CD. Since the CIT focused on specific problems related to the GFD, a comparison with a control group was not possible in this study.

Interestingly many of our findings in CD children/adolescents are similar to those found in CD adults by Sverker *et al*. using the same method of investigation [10]. Our study could present a report bias, given that many questionnaires were answered by the parents and not directly by the children (when younger than 8). However we could not find any noticeable differences in reported dilemmas between parents and children. Children with CD reported to experience dilemmas in three different “arenas” (contexts): meals at school, meals at home, food situations and meals outside home. Unlike adults, travel and purchase were not mentioned by anyone of the participants. This is not an unexpected finding, given that these arenas are usually managed by the patients’ parents.

In CD children we identified the same three main categories of dilemmas as experienced by adults: emotions, relationships and management of daily life. Children with CD described more emotions than adults, with strong feelings like anger, sadness or sense of diversity that could negatively impact their quality of life. Also social relationships were deeply influenced by CD and the GFD, but interestingly children with CD did not feel forgotten or neglected, probably because parents provided all their food-consumption needs. Management of daily life can be difficult for children and even more difficult for adolescents affected with CD, however we found only few dilemmas in this category. The influence of parents and the family environment played a primary role in children’s lives, and probably reduced dilemmas and difficult situations that could become more important later in life.

## 5. Conclusions

Our data clearly show that a part of the difficulties that children experience in coping with the GFD are not related to the quality of gluten-free food itself, that has improved over the last years, but could be influenced by other environmental factors, such as poor awareness of CD treatment in the general population and catering staff, and low availability of gluten-free food in most restaurants and cafeterias. These problems could be easily overcome by targeted information campaigns and staff training.

Our study shows that having a chronic illness like CD and following a GFD can negatively impact the QOL of affected children, a dimension that is not easily measured by standardized psycho-social questionnaires. A qualitative analysis of the psycho-social dilemmas, as performed by the CIT, provides information on many aspects of the difficulties that children with CD have to cope with, because of a limited food choice. These psychological and social aspects should be taken into account in the management of children and adolescents affected with CD.
